# Progressive Two-Stage Network for Low-Light Image Enhancement

**DOI:** 10.3390/mi12121458

**Published:** 2021-11-27

**Authors:** Yanpeng Sun, Zhanyou Chang, Yong Zhao, Zhengxu Hua, Sirui Li

**Affiliations:** 1 College of Electronic and Information Engineering, Shenyang Aerospace University, Shenyang 110136, China; huazhengxu@stu.sau.edu.cn (Z.H.); lisirui@stu.sau.edu.cn (S.L.); 2 Science and Technology on Altitude Simulation Laboratory, Mianyan 621700, China; yong_zhao@yeah.net

**Keywords:** image enhancement, two-stage network, residual dense network, attentional mechanisms

## Abstract

At night, visual quality is reduced due to insufficient illumination so that it is difficult to conduct high-level visual tasks effectively. Existing image enhancement methods only focus on brightness improvement, however, improving image quality in low-light environments still remains a challenging task. In order to overcome the limitations of existing enhancement algorithms with insufficient enhancement, a progressive two-stage image enhancement network is proposed in this paper. The low-light image enhancement problem is innovatively divided into two stages. The first stage of the network extracts the multi-scale features of the image through an encoder and decoder structure. The second stage of the network refines the results after enhancement to further improve output brightness. Experimental results and data analysis show that our method can achieve state-of-the-art performance on synthetic and real data sets, with both subjective and objective capability superior to other approaches.

## 1. Introduction

Images acquired under insufficient illumination are underexposed, resulting in images with uneven illumination having low visibility, invisible details and other visual degradation issues. Furthermore, this affects the applicability of the acquired images for subsequent advanced vision tasks, such as traffic monitoring, image segmentation, object detection, mechanical manufacturing, and target recognition and tracking [1,2]. In light of this, single-image enhancement as a basic low-level vision task has attracted increasing attention from computer vision researchers and artificial intelligence companies during the past few decades. The currently available enhancement algorithms can be broadly categorized into three groups, which includes (1) histogram equalization-based enhancement methods [3,4,5]; (2) Retinex theory-based enhancement methods [6,7,8,9]; or (3) deep learning-based enhancement methods [10,11,12,13,14,15,16,17].

Histogram equilibration-based enhancement methods enhance visual quality through the contrast of the image. These methods can achieve good results in processing images with dark foreground and background, but there are problems such as loss of details and color bias when some areas are over-enhanced. Banik et al. [3] proposed a method to enhance different types of low-light images using histogram equalization and illumination adjustment. Their method first applies histogram equalization to the V channel of the input low-light image after converting the color space from RGB to HSV. Then, the V-channel contrast is enhanced by adjusting the intensity of the low-light image and applying gamma correction. Kong et al. [4] proposed a method to enhance images with uneven illumination and low contrast. Their method derives bright enhancers and dark enhancers based on the resultant image produced by the mean filter. By applying the enhancers to the image, dark areas will be brighter and light areas will be darker. Sujee et al. [5] used the pyramidal layer histogram matching method to enhance the contrast of the image to extract maximum information. Their enhancement algorithm based on histogram equalization has a significant advantage in terms of processing time, but the overall image characteristics need to be considered in the brightness enhancement process, and acceptable results cannot be obtained when complex scenes are processed.

Retinex theory, first proposed by Land [6], principally employs visual constancy, which estimates the color of an object based on its reflection component. Based on the single-scale Retinex theory of Land [6], Jobson et al. [7] proposed a multiscale Retinex color recovery algorithm to perform image enhancement while maintaining image color consistency. Guo et al. [8] proposed a structure-aware a priori refined illumination map and computed it in the red, green, and blue channels to enhance the image. Fu et al. [9] applied the Retinex theory to propose a fusion-based low-exposure image enhancement method that can retain more details while enhancing the contrast. Although these methods achieve better results in some cases, the Retinex model still has limitations in the decomposition of reflectance and illumination, because this method cannot be applied to various scenes and adjusting the model parameters can easily lead to overexposure or underexposure of the resulting enhanced image.

With the rise of big data and deep learning, researchers have addressed the problem of low-illumination image enhancement by building deep learning network frameworks. Lore et al. [10] proposed a depth-based self-coding approach to enhance and denoise low-illumination images, which both improves the image brightness and avoids image overexposure. Li et al. [11] used a trainable, convolutional neural network (CNN), LightenNet, which takes weakly illuminated images as input and subsequently outputs their illumination maps to obtain enhanced images based on the Retinex model. Wang et al. [12] proposed a global illumination-aware and detail-preserving network. This architecture consists of two steps. First, The encoder and decoder network obtains fixed-size illumination predictions from a global perspective. Then, the convolutional network reconstructs the details using the illumination prediction and original input. Wei et al. [13] combined human vision theory and a convolutional neural network to decompose low-exposure images into reflectance and illumination maps, and used the enhancement network to increase achieve image brightness. Lv et al. [14] proposed a multi-branch, micro-light enhancement network (MBLLEN). The core idea is to extract rich features at different levels, enhance them by multiple sub-networks, and finally derive the output image by multi-branch fusion. In this way, image quality can be improved in all aspects. The method proposed by Wang et al. [15] did not directly train the mapping between low luminance images and normal luminance images, but rather estimated the mapping between images to illumination maps to enhance underexposed images. This method enhanced the network learning to adjust complex images. Zhu et al. [16] proposed a three-branch convolutional neural network (RRDNet) that decomposes the input image into three components: illumination, reflection, and noise. Pairs of datasets are not required as data drivers. It is updated by iteratively minimizing a specially designed loss function. Lim et al. [17] proposed a new low-light image enhancement method, Deep Laplace Restorer (DSLR), by exploiting the useful properties of Laplace pyramids in image and feature spaces. This method can recover global illumination and local details from the original input separately and combine them step by step in the image space. Although deep learning-based enhancement methods have shown significant performance improvements over traditional prior methods, the enhancement capability of these approaches is limited due to the fact that they only consider single-stage encode/decode structure. Therefore, this paper introduces an effective two-stage idea to further address the problem of insufficient enhancement of low-light images.

Drawing on the above analysis, this paper proposes a new low-light image enhancement network based on the progressive two-stage model, which takes the low-light image as input and outputs the enhanced image through the first-stage coder decoder structure and the second phase of the fine-grained network. The network not only considers the change of image from dark to light, but also performs image recovery from coarse to fine scale, which makes the enhanced image more detailed and brighter. The innovative features of this method are summarized as follows:

1. A two-stage image enhancement network was proposed, which grades the deep learning enhancement problem into two stages of coarsening and refinement for step by step processing and obtains preliminary enhancement results before refinement of specific scenes.
2. With the introduction of a two-stage lightweight network structure, enhancing complex scenes at night is more readily resolved, and the recovered images are richer in color, higher in sharpness and contrast, and with generally better visual quality.
3. The residual dense attention module is introduced into the network, which enhances feature extraction and recovers clear background images.

## 2. Proposed Method

In this section, we present in detail the proposed asymptotic two-stage model. It specifically describes the overall network architecture, attention mechanism, residual dense module and loss function.

### 2.1. Overall Network Architecture

**Figure 1:**
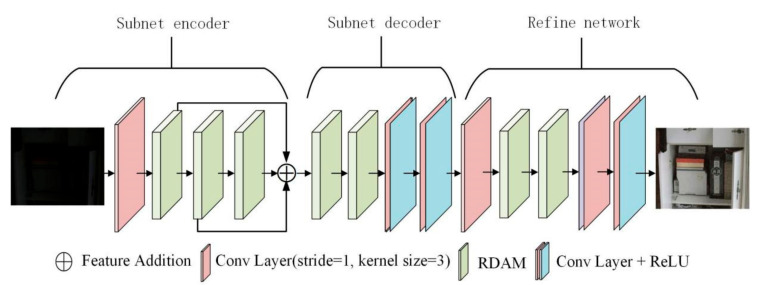
Overall network architecture.

To address the problems of texture detail loss and color distortion in existing algorithms, this paper proposes a progressive two-stage model for low-light image enhancement. Inspired by existing works [18,19], the model is integrated into an end-to-end trainable network that takes a low-light image as input and outputs the enhanced image through a first-stage encoder-decoder structure and a second-stage fine network. The overall network architecture is shown in Figure 1. In order to learn multi-scale features, the images output from the coder-decoder structure are passed to the fine-scale sub-network as additional information that can be used to refine the coarse-scale prediction of the previous stage. In addition, each level of the network is based on the residual dense attention module (RDAM) to obtain accurate and clear image estimation. The residual dense block (RDB) allows better utilization of information from the previous stage and facilitates feature learning at a later stage.

### 2.2. RDAM

**Figure 2:**
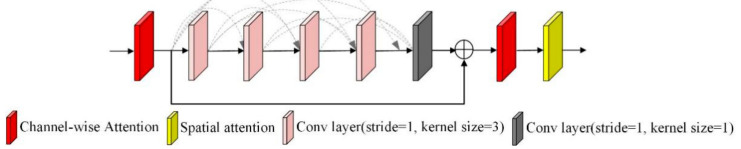
Residual dense attention module.

Network accuracy tends to saturate as its depth increases so that deep networks face the problem of significant performance degradation. In order to maximize the information flow between layers, we introduce the residual dense attention module, which contains two parts, the residual dense module and the attention mechanism, to solve the network performance degradation problem and correctly recover the image features. The specific network structure is shown in Figure 2.

#### 2.2.1. RDB

The residual dense module concatenates all matched feature maps and reuses the lower layer features in the higher layers to improve recovery accuracy. In this way, we preserve the forward propagated features and the outputs of each layer are obtained from operating on the previous results. This approach can effectively improve the propagation of features transmitted by hierarchical networks and promote feature reuse. The output of one residual dense module can directly access each layer of the next residual dense module, thus producing continuous state transmission. A residual dense network can be defined as follows:

(1) $$y_{l+1}=y_{l}+F\left(G_{d}\left(\left[y_{0},\cdot \cdot \cdot ,y_{d}\right]\right),\left[W_{0},\cdot \cdot \cdot ,W_{d}\right]\right)$$

Here, $y_{l}$ and $y_{l+1}$ are the *l* and *l* + 1 layers of the dense residual network, respectively; [*y*0,…, *yd*] and [*W*_0_,…, *W_d_*] are the parameters and feature information corresponding to all convolutional layers in the dense residual network, respectively. F is a residual network feature processing function; *G* represents a dense network.

Each convolutional layer in the residual dense module has access to all subsequent layers and passes the information that needs to be preserved. In our proposed network, first, the original image is convolved to extract low-level features and then passes through the residual dense modules, each of which is composed of five convolutional layers. The first four layers are used to increase the number of feature maps, the last layer is used to fuse these feature maps, and then the output is combined with the input of this residual dense module by the addition of the channel attention module and spatial attention module. Here, the input channel size and growth rate in each residual dense module are set to 2.

#### 2.2.2. Attention Mechanism

The attention mechanism enables the image processing network to assign different attention values to better focus on the features we are interested in and suppress the non-important features, which has important applications in image processing. In this paper, we add a channel-based attention module and a spatial attention module to the network to assign different channels and spaces to the features of different images to determine learning weights, This enables the network architecture to selectively perform feature extraction and avoid problems due to color distortion, over-enhancement, noise methods, and other issues.

**Figure 3:**
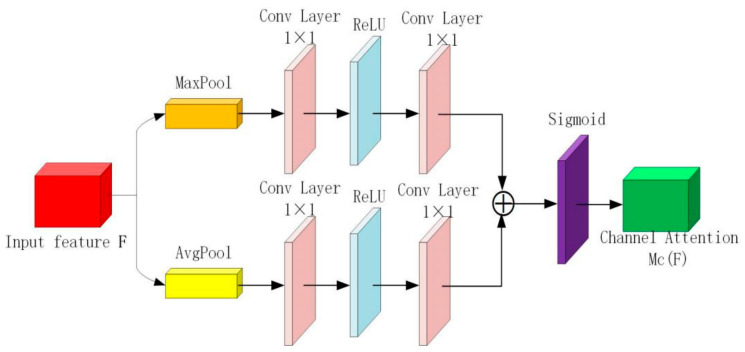
Channel attention module.

The channel attention module is shown in Figure 3. The channel attention mechanism can effectively determine the correlation between different channels and obtain the weight coefficients of each channel through network learning, thus reducing the learning of features such as noise, which is beneficial for enhancement of low illumination images. The channel attention map calculation can be defined as:

(2) $$W_{c}\left(F\right)=\sigma \left(\omega_{1}(P_{avg}(F))+\omega_{1}(P_{\max}(F))\right)$$

Here,F is the input characteristic; $\omega_{1}$ represents weights between layers of perceptrons; and $P_{avg}$ and $P_{\max}$ represent average pooling and maximum pooling, respectively.

**Figure 4:**
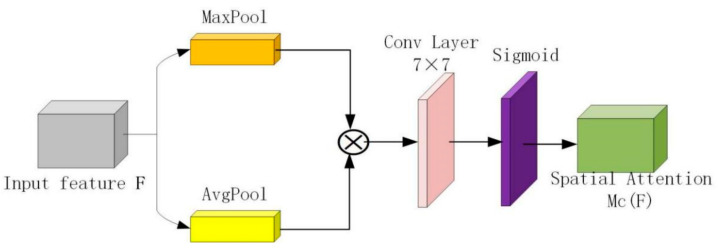
Spatial attention module.

The spatial attention module is shown in Figure 4. The spatial attention mechanism can transform the information on the image space to learn more representative features while weakening irrelevant background regions, thus improving the feature representation of key regions. Based on the spatial attention map scaled to 0~1, the formula can be expressed as:

(3) $$Ms\left(F\right)=\sigma \left(f^{7\times 7}\left(Concat\left[MaxPool\left(F\right);AvgPool\left(F\right)\right]\right)\right)$$

where σ denotes sigmoid function, and $f^{7\times 7}$ represents a conv layer of 7×7.

### 2.3. Two-Stage Network

#### 2.3.1. Phase I Network

For the first stage network structure, we use the encoder-decoder structure to obtain the preliminary enhanced image. The edge information of the image is one of the most basic features, and to ensure that it is not unnecessarily lost during the network convolution process, the edge map of the input image is calculated as a guide. First, the original input image is passed through a convolution kernel of 3, a step size of 1, and 16 convolution layers to extract the basic features of 16 layers; then, these basic features are passed through three RDB modules, and the output of each stage module is fused with multi-scale features in an additive manner; next, the extracted features are passed through two RDB modules and two basic modules, each of which consists of a 3 × 3 convolutional layer and a ReLU activation function; finally; the output is used as an enhanced image of the first stage coarse network, which will provide a clearer image for the second stage network.

#### 2.3.2. Phase II Network

After the first stage of the encoder-decoder network, the low-illumination image is directly restored to a sharper image, which still has incomplete details. Therefore, to further improve the quality of the enhanced images, we introduce a second-stage network that gradually refines the image details with essentially unchanged parameters. Since these two stages work in concert for image recovery, the enhanced image from the previous stage guides and facilitates detail recovery in the later stage. The enhanced image from the first stage is combined with the low-light image as the common input to the second stage. Similarly, we first extract a convolutional layer that passes through a convolutional kernel size of 3, a step size of 1, and 16 convolutional layers, and then two residual dense attention modules and the basic module to optimize the first stage enhancement results and further improve the visual quality of the output image.

### 2.4. Loss Function

To train the proposed network, we use the loss function as our optimization objective to evaluate the difference between the enhanced image and the real low-light image. The loss function L in this paper combines the MSE (mean square error) loss and SSIM perceptual loss and can be expressed as follows.

(4) $$L=L_{r}+\lambda L_{p}$$

In the formula, $L_{r}$ represents the mean square error loss; $L_{p}$ denotes the perceived loss; λ represents the weight factor regulating the two-loss functions and is set to 0.005 here.

The difference between the enhanced image $f_{i,j}$ and the ground-truth image $g{\left(I\right)}_{i,j}\;$ can be calculated using the MSE loss function as in the following equation:

(5) $$L_{r}=\frac{1}{HW}\overset{H}{\underset{i=1}{\sum }}\overset{W}{\underset{j=1}{\sum }}\left(f_{i,j}-g{\left(I\right)}_{i,j}\right)$$

Here I is the input real low-light image, and H, W are the width and height of the enhanced image, respectively.

The MSE metric may lead to problems such as loss of high frequency information and image smoothing. Thus, to improve visual quality we first trained the multi-scale features extracted from the deep neural network to quantitatively estimate the difference between the low-light image and the enhanced image. The generated image and the target image were subjected to VGG-16 feature maps. The perceptual loss of these two images after passing through the pooling layer is computed as:

(6) $$L_{p}=\frac{1}{HW}\sum_{i=1}^{H}\sum_{j=1}^{W}{\left(\varphi {\left(I_{H}\right)}_{i,j}-\varphi {\left(I_{L}\right)}_{i,j}\right)}^{2}$$

In this equation, H and W represent the length and width respectively of the feature map. $I_{H}$ represents the output enhanced image; $I_{L}$ is a clear image that needs to be compared.

## 3. Experimental Results and Analysis

To illustrate the effectiveness and robustness of the network, qualitative and quantitative analytical comparison experiments were performed on synthetic and real data, respectively, comparing and analyzing them comprehensively with five existing mainstream image enhancement algorithms, LightenNet [11], MBLLEN [14], Retinex-Net [13], RRDNet [16], and DSLR [17], as mentioned previously. Peak signal-to-noise ratio (PSNR) and structural similarity (SSIM) data were chosen to quantify the objective evaluation metrics. The codes used are from official sources and were trained and tested on the same dataset and with the same experimental conditions and parameters.

### 3.1. Data Sets

At present, the most commonly used public datasets for image enhancement training include BrighteningTrain, DPED, SID, LOL, etc. In this paper, the database used for the image enhancement experimental training network is the LOL [13] public dataset, which contains 500 low-exposure images and corresponding normal-exposure images, each of size 400 × 600 pixels. Here, 485 image pairs from this database were used for training and 15 image pairs were used for testing.

### 3.2. Parameters Setting for the Experimental Environment

All of our experiments were conducted on the Ubuntu 18.04 operating system with Python as the programming language, Pytorch as the deep learning framework, Nvidia 1080Ti GPU as the graphics card, and 8GB of RAM. In total 485 image pairs in LOL were trained with a period of 50 and accelerated using the Adam optimizer, with β1 and β2 set to 0.9 and 0.999, respectively, and the loss function set to 0.005. The initial learning rate was set to 0.001, the Batch Size to 2, and the learning rate was halved every 20 cycles.

### 3.3. Objective Indicators

To objectively evaluate image quality, this paper employs the two most commonly used indicators: peak signal-to-noise ratio (PSNR) and structural similarity (SSIM), for measurement.

PSNR is the most widely used objective evaluation index for full-reference image quality, where a larger value of PSNR indicates better image quality. PSNR is calculated as follows:

(7) $$\mathrm{PSNR}=10\times \mathrm{lg}\left(\frac{{\left[f_{\max}-f_{\min}\right]}^{2}}{MSE}\right)=10\times \mathrm{lg}\left(\frac{{\left[255-0\right]}^{2}}{MSE}\right)$$

(8) $$MSE=\frac{1}{M\times N}\sum_{x=1}^{M}\sum_{y=1}^{N}{\left[f^{\prime }(x,y)-f(x,y)\right]}^{2}$$

where M, N denote the height and width of the image respectively, and MSE denotes the mean square error of the image. $\left(x,y\right)$ represents image pixel coordinates.

SSIM measures image similarity in terms of brightness, contrast, and structure, and is also a fully-referenced objective evaluation index, with value ranging from 0 to 1. The larger the value, the less distortion and the higher the degree of image similarity.

(9) $$\mathrm{SSIM}\left(x,y\right)=l\left(x,y\right)\cdot c\left(x,y\right)\cdot s\left(x,y\right)$$

In the formula, l represents the image brightness, c the contrast and s the structure.

### 3.4. LOL Results Analysis of Open Data Sets

**Figure 5:**
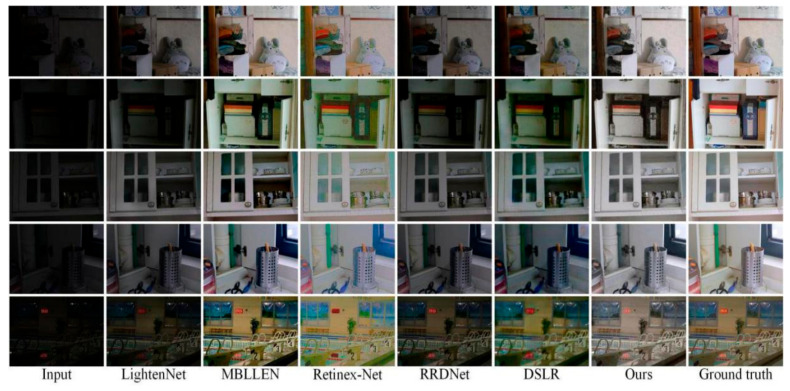
Image enhancement results of different algorithms.

We will show the final effect of this method on enhancing some images in the LOL data set to illustrate the effectiveness of this algorithm. The subjective effect is the most natural feeling of the human eye. Figure 5 shows the results obtained by our method and the other five methods. We can see that the LIME algorithm can restore the details, but it produces the phenomenon of overexposure; the Retinex-Net algorithm has serious color distortion, and the overall brightness of the image is low; the GLAD algorithm is more effective in brightness enhancement, but there is color distortion. KinD algorithm is more effective than the other three algorithms in enhancement. More effective get more natural results, but also appear the phenomenon of color distortion. Compared with these five algorithms, our algorithm realizes the brightness enhancement of the image, the color of the image is more natural, the texture of the problem is more detailed, and the outline is clearer, and the result is closest to the real image.

In addition to subjective effects, this paper also uses the objective indicators introduced in Section 2.3 to compare the methods. Table 1 shows the average value of the objective index PSNR and SSIM of 15 images enhanced by this algorithm and five other algorithms. Through the results of Table 1, the PSNR value of this algorithm exceeds 23 dB, SSIM, and the PSNR value of the other five algorithms is higher than that of 0.8, which shows that the algorithm can restore the detail features while ensuring the output brightness. Make the enhanced image closer to the real situation.

### 3.5. Natural Real Image Test Results

**Figure 6:**
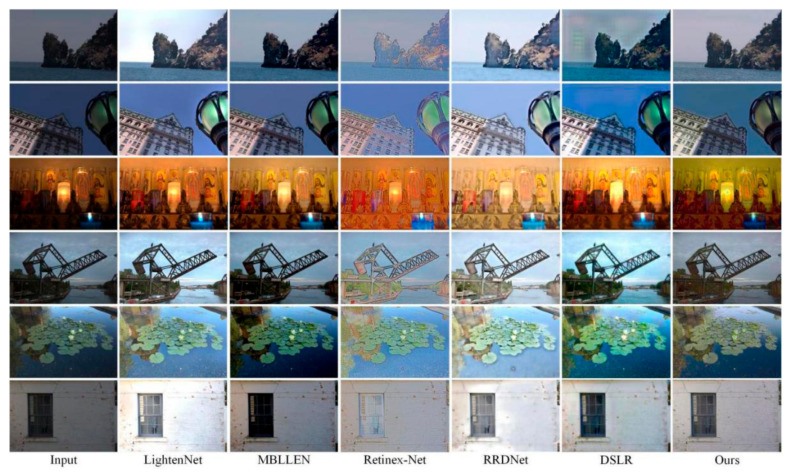
Comparison of different algorithms for real image results.

In order to further verify the effectiveness of this method for real images in nature, real low-light images are selected for comparative experiments. Figure 6 shows one of the contrasting scenarios.

We note that the output performance is consistent with the results of the above synthetic dataset. Specifically, the Retinex-Net and DSLR algorithm have partial distortion and noise amplification in color, and the overall brightness of the images are low. The enhancement results of LightenNet algorithm are very competitive, but unfortunately, it is over enhanced, which may be unacceptable in real-world applications. Compared with these five algorithms, our algorithm enhances the brightness and suppresses the noise, which makes the image edge features and details more complete, and the overall tone is more in line with people’s intuitive visual feelings. Through the above experiments, our enhancement results are more impressive and beneficial to real-world applications.

## 4. Conclusions

In this paper, a progressive two-stage model ground low-light image enhancement algorithm is proposed consisting of an encoder-decoder structure and a second-stage refinement network. The first-stage network extracts the multiple scale features of the image through the encoder and decoder structures, and the second-stage network further enhances the image quality by additional local refinement. The experimental results and data analysis show that the network in this paper has superior performance to other algorithms for visual quality after image enhancement. It is worth noting that the subjective evaluation of our proposed algorithm needs to be further improved. Further, this network provides a reference image processing scheme for scene tasks such as enhancing low light images. The next step is to consider using the proposed lightweight model on other image restoration problems.

## Figures and Tables

**Table 1:** Objective indicators of enhancement results of different algorithms in LOL data sets.

Methods	LightenNet	MBLLEN	Retinex-Net	RRDNet	DSLR	Ours
PSNR/dB	11.85	20.23	19.27	13.00	17.16	22.14
SSIM	0.6023	0.8233	0.5792	0.6646	0.7562	0.8352

## Data Availability

The available online of LOL dataset in this paper is https://daooshee.github.io/BMVC2018website/.
